# Growth Performance, Immuno-Oxidant Status, Intestinal Health, Gene Expression, and Histomorphology of Growing Quails Fed Diets Supplemented with Essential Oils and Probiotics

**DOI:** 10.3390/vetsci12040341

**Published:** 2025-04-07

**Authors:** Rania El Sayed Mahmoud, Ahmed Ateya, Hossam Gadalla, Hanan M. Alharbi, Khairiah M. Alwutayd, Eman M. Embaby

**Affiliations:** 1 Department of Nutrition & Clinical Nutrition, Faculty of Veterinary Medicine, Mansoura University, Mansoura 35516, Egypt; ranianutrition@mans.edu.eg; 2 Department of Development of Animal Wealth, Faculty of Veterinary Medicine, Mansoura University, Mansoura 35516, Egypt; 3 Department of Clinical Pathology, Faculty of Veterinary Medicine, Mansoura University, Mansoura 35516, Egypt; gadallaha@mans.edu.eg; 4 Department of Biology, College of Science, Princess Nourah bint Abdulrahman University, P.O. Box 84428, Riyadh 11671, Saudi Arabia; hmalharbi@pnu.edu.sa (H.M.A.); kmalwateed@pnu.edu.sa (K.M.A.); 5 Department of Physiology, Faculty of Veterinary Medicine, Mansoura University, Mansoura 35516, Egypt; emanmohamed@mans.edu.eg

**Keywords:** quails, growth, essential oils, probiotics

## Abstract

This study aimed to evaluate how natural dietary supplements, including essential oils (EOs) and probiotics, influence the growth performance, carcass traits, serum components, gut function, gene expression, and jejunal histomorphology of growing quails. A total of 240 unsexed 7-day-old growing Japanese quails were randomly assigned to four experimental groups (n = 60 per group), with each group further divided into six replicates (10 quails per replicate). The control group (S_0_) received a basal diet without incorporating any additives, while the experimental groups were supplemented with (i) essential oils (S_1_); (ii) probiotics (S_2_); or (iii) a mixture of EOs and probiotics (S_3_) at a level of 1.5 kg/ton and 0.55 g per kg diet, respectively, and the ratio of the mixture of EOs and probiotics was approximately 2.73:1. The results showed that, from 7 to 35 days of age, S_3_ quails showed increased growth performance, carcass weight, and serum total protein with a decreased lipid profile, outperforming the individual supplementation of either additive (*p* < 0.05). Importantly, EOs or probiotics enhanced immune-antioxidant status in growing quails, particularly those who were fed both EOs and probiotics, showing significantly increased levels of the serum immune parameters IgY and IgM as well as boosting T-AOC, SOD, and GPx levels when MDA content was lowered compared to S_0_ quails (*p* < 0.05). Additionally, in quails fed a mixture of EOs and probiotics, the primary pro-inflammatory factors TNF-α, IL-1β, and IL-6 were downregulated, and the anti-inflammatory factors TGF-β and IL-10 were elevated compared to the S_0_ group (*p* < 0.05). In this context, there was a notable increase in growth (IGF-I, myogenin, and AvUCP), immunological (IL-2, IL-4, IL-6, and AVBD), antioxidant (SOD, CAT, GPx, and ATOX1), and intestinal absorption (VEGF, MUC2, GLUT2, calbindin, and FABP6) markers in quails supplemented with EOs and/or probiotics when compared to the control group (*p* < 0.05). The combination of EOs and probiotics had the most noticeable impact on the markers’ expression patterns compared to either additive alone (*p* < 0.05). Consistent with our results, quails given both EOs and probiotics showed significantly greater villi in terms of height and width when compared to the control group in intestinal histomorphology, the primary measure of intestinal wellness. In conclusion, quail diets could benefit from the use of EOs or probiotics as natural growth promoters since they improve growth performance, blood parameters associated with protein and lipid profiles, immune-antioxidant status and inflammation, and marker gene expression profiles of growth, immune, antioxidant, and intestinal absorption.

## Simple Summary

The Japanese quail has grown in importance in the poultry sector and has contributed to improving human nutrition by delivering high-quality organic meat and eggs. The use of natural beneficial substances in quail diets, such as essential oils and/or probiotics, promotes nutritional digestibility, maintains intestinal villus integrity, boosts productive performance, and improves the oxidative-immune status of growing quails. The findings of this study support the use of natural alternatives, such as essential oils and probiotics, either individually or in combination, to achieve optimal growth performance in terms of live body weight and feed conversion ratio and improve immune-antioxidant status in addition to the intestinal health and absorption that improve nutrient utilization in growing quails. Furthermore, a combination of essential oils and probiotics has the potential to function synergistically and has been shown to be even more effective than each alone in growing quails.

## 1. Introduction

The Japanese quail, which produces premium organic meat and eggs, has grown in importance in the poultry sector and contributed to bettering human nutrition [1]. Enhancing poultry production requires a better understanding of how diet affects overall health, which can be achieved through advanced nutritional approaches [2]. Developing sustainable solutions that satisfy the requirements of commercial production is crucial as the demand to identify antibiotic substitutes with equivalent economic benefits grows [3].

The primary concern regarding the diet of poultry birds is the impact of different feed additives on gut health [4]. While some feed additives, such as essential oils, herb extracts, electrolytes, prebiotics, probiotics, and organic acids, have beneficial effects, others, such as toxic chemicals and aflatoxin, have negative effects that affect the health and productivity of birds [5]. Adding feed additives to the diet maximizes the utilization of feed, increases feed conversion efficiency, and ultimately improves the growth and health of birds [6].

Recently, probiotics, prebiotics, synbiotics, postbiotics, polysaccharides, organic acids, enzymes, and essential oils have been explored as feed additives with great potential in the poultry industry [7]. Probiotics have recently been recognized as beneficial nutritional supplements in the poultry industry, providing an effective alternative to antibiotics. These multifunctional feed additives are often combined with other supplements to enhance productivity and support overall poultry health [5,6]. Probiotics with strains like *Lactobacillus* spp., *Bifidobacteriumbifidum*, and *Enterococcus faecium* have been shown to benefit intestinal microbiota. They enhance the structure of the intestinal mucosa, promoting better gut health and more efficient nutrient absorption in birds [8].

Essential oils (EOs), also known as volatile oils, are natural compounds extracted from different plant parts [9]. High-quality essential oils are characterized by their aromatic compounds and are free from chemicals, additives, and synthetic substances. These oils are widely recognized for their therapeutic and health benefits. They exhibit antimicrobial properties, support lipid metabolism, promote gastrointestinal health, and possess antioxidant and anti-inflammatory effects [10]. Carvacrol, capsaicin, and thymol powder are examples of essential oils proven to enhance broiler performance and carcass production when added to the diet [11]. For instance, Peng et al. [12] reported an improved feed conversion ratio when oregano leaves were included in poultry diets.

Based on the well-documented benefits of EOs and probiotics in poultry, we hypothesized that the use of EOs and probiotics would offer superior results compared to using them individually to optimize feed efficiency, growth rate, and immune regulation and enhance intestinal health and function. Therefore, this study aimed to evaluate whether EOs, probiotics, or their combination could act synergistically in growing quails and to explore the possible underlying mechanisms.

## 2. Materials and Methods

Research was conducted in the experimental unit of the Nutrition and Clinical Nutrition Department at the Faculty of Veterinary Medicine, Mansoura University. This study was approved by the research ethics Committee, Faculty of Veterinary Medicine, Mansoura University, Egypt, registration code number (MU-ACUC; VM.R.25.01.202).

### 2.1. Investigated Growth Promoters

The experiment’s growth promoters came from veterinary medicine businesses in the local Egyptian market. The manufacturer’s recommended dosage for each growth promoter was followed. The growth promoters were applied as a powder.

#### 2.1.1. Orego-Stim-Powder Compounds

Each 1 kg contains Oregano oil 50,000 mg, Carvacrol 36,000 mg, Thymol 500 mg, Diatomaceous earth 100,000 mg, and carrier calcium carbonate up to 1 kg. The data on the composition and dose of Orego-Stim-Powder have already been presented in the pamphlet given from Dakahlia Poultry Company, Mit Ghamr, Egypt. The Orego-Stim-Powder was manually weighed according to the recommended dosage (1.5 kg per ton of feed) and thoroughly mixed with the basal diet using a mechanical feed mixer to ensure uniform distribution. This process was repeated for each batch of freshly prepared feed to maintain consistency and avoid any segregation of ingredients.

#### 2.1.2. Probiotic Compound (Strong-G)

The probiotic compound contained a synergistic blend of betaine and the lactic acid producing bacteria *A. oryzae*, *E*. *faecium*, *B. bifidum*, *L. planterum*, *L. acidophilus*, and *B. subtillis* at a dose of 0.55 gm/kg from the diet according to the producing company (plexo pharm). The probiotics were kept in a cool, dry place at temperatures below 25 °C, protected from moisture and direct sunlight. To ensure stability and prevent degradation, the experimental diets were freshly prepared each week.

### 2.2. Experimental Site

The quails were housed in a partially enclosed rearing system with an autumnal natural photoperiod of 11–12 h, a room temperature of 25–30 °C, and a relative humidity of 55–70% within the Department of Nutrition and Clinical Nutrition at the Faculty of Veterinary Medicine, Mansoura University. A clean and hygienic atmosphere, adequate lighting, a water supply, ventilation systems with fans and air suction, and other necessities were all present in the facility to meet the demands of each trial group. The control diet was created to satisfy the nutritional requirements of growing Japanese quails in accordance with NRC [13] requirements, and the ingredients and nutrient composition of the control diet are shown in Table 1.

### 2.3. Quails, Treatments, and Experimental Design

A total of 240 unsexed quails were raised together from hatching to seven days of age, and they were provided a basal control diet for the first seven days. From 1 to 3 days of age, the temperature was maintained at 34 °C (floor level); from 4 to 7 days, it was maintained at 31 °C; from 8 to 14 days, it was maintained at 28 °C; and ultimately, it was progressively lowered to room temperature (24–26 °C). At seven days of age, the quails were divided into four treatment groups; the control group (S_0_) received a basal diet without incorporating any additives, while the experimental groups were supplemented with (i) EOs (S_1_); (ii) probiotics (S_2_); or (iii) a mixture of EOs and probiotics (S_3_) at a level of 1.5 kg/ton and 0.55 g per kg diet, respectively, and the ratio of the mixture of EOs and probiotics was approximately 2.73:1. Each group was further subdivided into six replicates, with 10 birds per replicate. Throughout the 4-week study, the quails had continuous access to feed and water. To preserve ingredient stability and prevent evaporation or degradation, the experimental diets were freshly prepared each week.

### 2.4. Growth Performance and Carcass Traits

At the start of the experiment, each quail was weighed individually, and their weights were recorded weekly. Using these measurements, weekly body weight gain (BWG), average feed intake (FI), and feed conversion ratio (FCR) were assessed. The FCR was determined with the following formula: FCR = Total feed intake (g)/Body weight gain (g). To evaluate carcass traits, one bird per replicate was selected at the end of the experiment (35 days old), ensuring it was closest to the average body weight within the cage, and euthanized humanely through cervical dislocation. Euthanasia of the quails was performed according to the American Veterinary Medical Association (AVMA) guidelines on euthanized animals, 2020 Edition. The birds were then eviscerated. Feathers and blood were removed to determine the carcass weight, and the head, feet, and belly fat were removed to determine the eviscerated weight. To determine their relative proportions, the weights of the internal organs and carcass were expressed as a percentage of live body weight (LBW) using the following formulas:

Internal Organ Weight (%) = (Organ weight (g) ÷ LBW (g)) × 100

### 2.5. Serum Biochemical Indices

At the end of the experiment, serum samples were collected by centrifuging approximately 2 mL of pre-euthanasia blood drawn from the wing vein of six quails per treatment. The blood was placed in tubes without anticoagulant and spun at 3400× *g* for 9 min using a SIGMA 4-15 Lab Centrifuge (Godenbergstrasse 523714 Bad Malente, Malente, Germany). The clear serum was carefully separated, transferred to Eppendorf tubes, and stored in a deep freezer at −20 °C until chemical analysis. Serum levels of total protein (TP), albumin (ALB), calculated globulin (GLB), blood urea nitrogen (BUN), total cholesterol (TC), triglycerides (TG), high-density lipoprotein (HDL), and low-density lipoprotein (LDL) were determined using specific kits from Sentinel Diagnostics (Milano, Italy) or CAL-TECH Diagnostics, Inc. (Chino, CA, USA). Measurements were performed calorimetrically using a Spectronic 1201 spectrophotometer (Milton Roy, Ivyland, PA, USA).

According to the manufacturer’s guidelines, a quail-specific ELISA kit (R & D, Minneapolis, MN, USA) was utilized to assess serum levels of immunoglobulins (IgY and IgM). According to [14], redox status indicators, including superoxide dismutase (SOD), glutathione peroxidase (GPx), malondialdehyde (MDA), and total antioxidant capacity (T-AOC), were evaluated. Additionally, serum concentrations of pro-inflammatory cytokines (interleukin-1β (IL-1β), interleukin-6 (IL-6), and tumor necrosis factor-α (TNF-α)) and anti-inflammatory molecules (transforming growth factor beta (TGF-β) and interleukin-10 (IL-10)) were measured using commercial ELISA kits from MyBioSource (San Diego, CA, USA). All analyses were performed following the manufacturer’s instructions.

### 2.6. Quantitative Real-Time PCR

Total RNA was isolated from quail muscle, spleen, liver, and intestine tissues using the Trizol reagent, following the manufacturer’s guidelines (Direct-zol™ RNA MiniPrep, Catalog No. R2050). The concentration and purity of the extracted RNA were assessed using a Nanodrop UV–Vis spectrophotometer (Q5000, Quawell Tech nology, San Jose, CA, USA), and RNA integrity was verified through gel electrophoresis. Complementary DNA (cDNA) was synthesized for each sample according to the manufacturer’s instructions (SensiFast™ cDNA Synthesis Kit, Bioline, Catalog No. Bio-65053). The reaction mixture (20 μL) consisted of 1 μg of total RNA, 4 μL of 5× TransAmp buffer, 1 μL of reverse transcriptase, and DNase-free water to a final volume of 20 μL. The reaction was conducted in a thermal cycler under the following conditions: primer annealing at 25 °C for 10 min, reverse transcription at 42 °C for 15 min, and enzyme inactivation at 85 °C for 5 min. The synthesized cDNA samples were stored at 4 °C. Real-time PCR was performed to assess the relative mRNA expression levels of genes associated with growth (IGF-I, myogenin, AvUCP) and immune function (IL-2, IL-4, IL-6, AVBD), antioxidant defense (SOD, CAT, GPx, ATOX1), and intestinal health (VEGF, MUC2, GLUT2, calbindin, FABP6). The sequences of the primers utilized are provided in Table 2. β-actin was used as the housekeeping gene for normalization. The reaction mixture (20 μL) included 10 μL of 2× SensiFast SYBR, 3 μL of cDNA, 5.4 μL of distilled water (H₂O), and 0.8 μL of each primer. The 2^−ΔΔCt^ method [15] was used to quantify the relative gene expression in each sample, with β-actin serving as the reference gene.

### 2.7. Intestinal Histomorphology

Jejunum tissue samples were obtained from 12 slaughtered quails per group within 30 min post-slaughter and preserved in 10% neutral buffered formalin for histopathological examination. The preserved tissues were embedded in paraffin, sectioned into 5 μm slices, and stained with hematoxylin and eosin (H&E) [16]. The jejunal slides were analyzed using a 10× eyepiece on an Olympus BX41 microscope (Olympus, New York, NY). Images were captured with a DVC 1300C color digital camera attached to the microscope. Intestinal villous height (VH, μm), villous width (VW, μm), and crypt depth (CD) were assessed according to the method outlined in [17]. The VH/CD ratio was determined, and all morphometric measurements were conducted by a pathologist on jejunal sections with vertically aligned villi using ImageJ version 1.54 software (http://imagej.en.softonic.com, accessed on 22 March 2025 ) for image analysis.

### 2.8. Statistical Analysis

All data were analyzed statistically using one-way ANOVA, followed by Tukey’s post hoc multiple range test following normality verification via Shapiro–Wilk and Levene’s tests, utilizing GraphPad Prism version 8.0 (GraphPad Software, Inc., San Diego, CA, USA). Mean values and the standard error of the mean (mean ± SEM) were calculated for each parameter, with differences considered statistically significant at *p* < 0.05.

## 3. Results

### 3.1. Performance Indices

As illustrated in Table 3, there were no notable variations in IBW among the experimental groups, while the statistical analysis confirmed notable variations between the groups (*p* < 0.05) in performance indices. The combination of EOs and probiotics showed the most pronounced effect, outperforming individual supplementation with either additive. This marked improvement suggests a synergistic effect between the two additives, resulting in superior growth performance. The control group displayed the lowest rate of growth indices. The groups of quails taking EOs or probiotics did not differ significantly from each other in growth performance indices.

### 3.2. Characteristics of the Carcass and Internal Organs

The statistical analysis revealed a significant improvement in carcass yield among quails fed a combination of essential oils and probiotics compared to the control group. However, no significant differences (*p* > 0.05) were detected among the experimental groups in the relative weights of internal organs, such as the heart, gizzard, liver, and spleen (Table 4).

### 3.3. Blood Constituents

As indicated in Table 5, the levels of serum TP were considerably higher in the S_3_ quails fed a mixture of EOs and probiotics than those in the other quails under study (*p* < 0.05). In the meantime, there were non-significant variations in the ALB and GLB values in response to the dietary supplements (*p* > 0.05). Furthermore, the supplemented quails fed a combination of EOs and probiotics showed a substantial drop in BUN levels in their sera in contrast to the other experimental groups (*p* < 0.05). Regarding the lipid profile, our results demonstrated that giving quails a diet enriched with a blend of EOs and probiotics considerably decreased blood TG, TC, and LDL when compared to the other quails pursuant to this study. Nonetheless, the quails’ HDL levels dramatically rose when fed a diet that included both EOs and probiotics (*p* < 0.05).

The impact of dietary fortification with EOs and/or probiotics on the quails’ blood immunity, redox state, and inflammatory status is shown in Table 6. Regarding the current findings, when compared to the quails fed a control diet only, the experimental quails’ serum levels of IgY and IgM rose when they were supplemented with EOs or probiotics. Interestingly, the treated quails that were fed diets that included both probiotics and EOs and probiotics alone showed noticeably higher levels of Ig (*p* < 0.05). In terms of redox status, our analyses showed that incorporating EOs and/or probiotics improved the antioxidative state of the quails under study. The quails fed a mix of EOs and probiotics had elevated blood levels of T-AOC, SOD, and GPx and the lowest levels of MDA, a significant byproduct of lipid peroxide, when compared to the other groups under study (*p* < 0.05). With respect to pro- and anti-inflammatory cytokines, the quails given a combination of EOs and probiotics and probiotics alone showed anti-inflammatory beneficial properties in growing quails by the noticeable decreases in the levels of IL-1β, IL-6, and TNF-α and rises in the levels of TGF-β and IL-10 in comparison with the control group (*p* < 0.05).

### 3.4. Growth, Immune, Antioxidant, and Intestinal Absorption Marker Gene Expression Profiles

**Figure 1:**
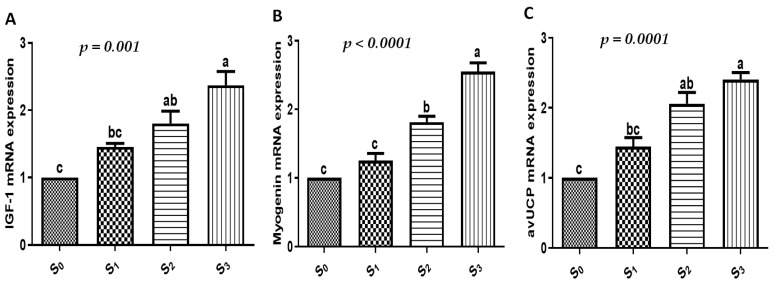
The effect of EOs, probiotics, or their combination on growth-related genes of growing quails. (**A**–**C**) mRNA expressions of growth-related genes (IGF-I in the liver and myogenin and AvUCP in the muscles) of our studied quails. Results are shown as mean ± SEM. Different small alphabetical letters mean significant when *p* < 0.05. S_0_: control diet with no supplements, S_1_: control diet supplemented with EOs, S_2_: control diet supplemented with probiotics, S_3_: control diet supplemented with a combination of EOs and probiotics.

**Figure 2:**
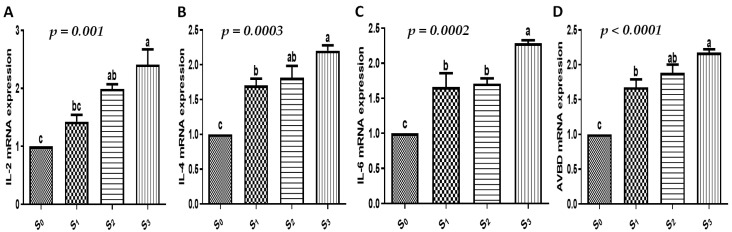
The effect of EOs, probiotics, or their combination on immune-related genes of growing quails. (**A**–**D**) mRNA expressions of immune-related genes (IL-2, IL-4, IL-6, and AVBD) in the spleen of our studied quails. Results are shown as mean ± SEM. Different small alphabetical letters mean significant when *p* < 0.05. S_0_: control diet with no supplements, S_1_: control diet supplemented with EOs, S_2_: control diet supplemented with probiotics, S_3_: control diet supplemented with a combination of EOs and probiotics.

**Figure 3:**
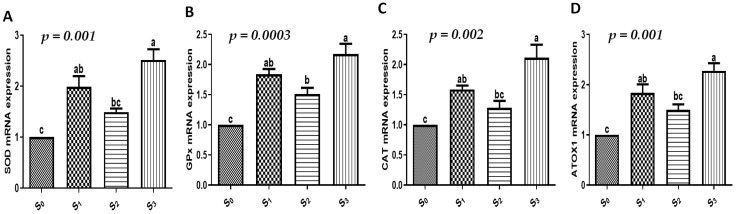
The effect of EOs, probiotics, or their combination on antioxidant-related genes of growing quails. (**A**–**D**) mRNA expressions of antioxidant-related genes (SOD, GPx, CAT, and ATOX1) in the liver of our studied quails. Results are shown as mean ± SEM. Different small alphabetical letters mean significant when *p* < 0.05. S_0_: control diet with supplements, S_1_: control diet supplemented with EOs, S_2_: control diet supplemented with probiotics, S_3_: control diet supplemented with a combination of EOs and probiotics.

**Figure 4:**
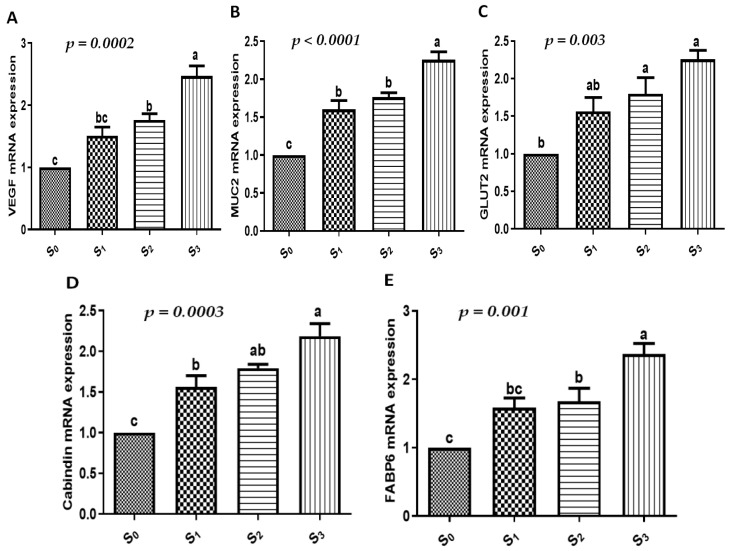
The effect of EOs, probiotics, or their combination on intestinal absorption-related genes of growing quails. (**A**–**E**) mRNA expressions of intestinal absorption-related genes (VEGF, MUC2, GLUT2, calbindin, and FABP6) in the intestines of our studied quails. Results are shown as mean ± SEM. Different small alphabetical letters mean significant when *p* < 0.05. S_0_: control diet with no supplements, S_1_: control diet supplemented with EOs, S_2_: control diet supplemented with probiotics, S_3_: control diet supplemented with a combination of EOs and probiotics.

The present investigation assessed the gene expression profiles for markers of growth, immune function, antioxidants, and intestinal function. When the quails were supplemented with EOs and/or probiotics, there was a significant increase in growth (IGF-I, myogenin, and AvUCP, Figure 1), immunological (IL-2, IL-4, IL-6, and AVBD, Figure 2), antioxidant (SOD, CAT, GPx, and ATOX1, Figure 3), and intestinal absorption (VEGF, MUC2, GLUT2, calbindin, and FABP6, Figure 4) markers compared to the control group (*p* < 0.05). The combination of EOs and probiotics had the most apparent influence on the expression pattern of the examined markers compared to either additive alone (*p* < 0.05).

### 3.5. Histological Examinations

**Figure 5:**
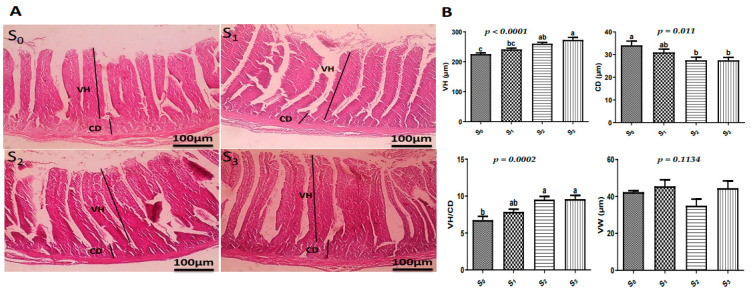
(**A**) Microscopic pictures of H&E-stained jejunal sections revealing normal histology in all groups with markedly increased VH in S_3_ (X: 100 bar 100). (**B**) Bars represent statistical analysis of more significantly increased VH, significantly decreased CD, and significantly increased VH/CD ratio in jejunal sections in S_3_ compared to the other studied groups. Different small alphabetical letters mean significant when *p* < 0.05. S_0_: control diet with no supplements, S1: control diet supplemented with EOs, S_2_: control diet supplemented with probiotics, S_3_: control diet supplemented with a combination of EOs and probiotics.

Microscopic examination showed normal jejunal mucosa, submucosa, and muscular layer in the control group and the other experimental groups (Figure 5A). Significantly higher jejunal VH and VH/CD with significantly lower CD were recorded in the birds in S_2_ and S_3_ (*p* < 0.05) than in S_0_ and S_1_ (Figure 5B). Non-significant changes were recorded in VW.

## 4. Discussion

The results of this study asserted significant improvements in FBW and BWG when EOs and probiotics were included in the diet. The combination of these supplements yielded the highest FBW and BWG, outperforming the groups receiving either supplement individually or the control diet. This reflects the potential of natural feed additives to enhance growth performance by improving gut health and nutrient utilization. This illustrates the possibility of EOs and probiotics working in concert to maximize growth performance. As noted in studies by Santos et al. [18], EOs, such as thymol and carvacrol, can improve gut morphology, support digestive processes, and enhance nutrient absorption. This could explain the significant growth performance improvement observed in the EOs group compared to the control group. Similarly, Gholami-Ahangaran et al. [11] found a positive effect on the performance of broilers by adding EOs to the diet. Moreover, Kürekci et al. [19] stated that some studies have demonstrated the positive impact of dietary supplementation with EOs or plant-based compounds on the growth performance of quails and poultry. However, research by Cetin et al. [20] revealed that supplementing quail diets with rosemary oil had no significant effect on performance indices.

The results of our study align with the findings by Saeed et al. [21], who demonstrated that probiotics enhanced VH and reduced CD, promoting better nutrient absorption and growth. The improved performance in the probiotics group highlights their potential as a dietary supplement for maximizing performance. With the same concept, Al-Younes et al. [22] concluded that incorporating probiotics in broiler diets improved broilers growth parameters. Additionally, El-Sayed et al. [23] demonstrated that phytobiotics, such as EOs, and probiotics exhibited a significant increase in performance indices in broiler chickens compared to the control group. The combination of EOs and probiotics demonstrated the most significant improvement in performance, indicating a synergistic effect. The complimentary mechanisms of these supplements might be the cause of this synergy. By fostering an environment in the gut that prevents the growth of dangerous bacteria, EOs facilitate the colonization and growth of probiotics. Probiotics may thereby increase the bioavailability of EOs’ active ingredients, enhancing their advantages. These findings are consistent with the work of Chen et al. [24], who indicated enhanced growth performance and feed conversion efficiency when phytogenics and probiotics were combined. EOs reduce oxidative stress and inflammation, while probiotics enhance nutrient absorption and maintain a balanced microbiota. Together, these mechanisms optimize energy use, resulting in a better growth outcome. The FCR, an indicator of feed efficiency, was significantly improved in all treatment groups compared to the control diet, with the combination of EOs and probiotics showing the lowest FCR. This result reflects better nutrient utilization and energy efficiency, attributed to the complementary mechanisms of these additives. EOs enhanced the colonization and activity of probiotics by reducing gut pathogens, while probiotics amplified the bioavailability of EO components. Such interactions are consistent with the findings from Yang [25], who reported that combining probiotics with phytogenics significantly reduced FCR in livestock. Similarly, Hussein et al. [26] demonstrated that incorporating probiotics and photobiotics, either individually or together, into broiler diets enhances growth performance and supports intestinal health in broiler chicks.

The major indicator of gut health, intestinal morphology, may be connected to the positive impacts of the growth promoters under investigation on output [27]. Villus height, width, and density in a certain intestinal area are some of the aspects that define the small intestine’s functional state. An increase in the total luminal villus absorptive area may result from the extension of jejunal villi and the enhanced VH-to-CD ratio seen in the current study for either EOs, probiotics, or a combination of both. This could then lead to adequate digestive enzyme action and increased nutrient absorption at the villus surface, which would improve FCR and BWG [28]. In a comparable manner, a study by Viveros et al. [29] clarified that the elevated expression of brush boundary enzymes is linked to the rise in villus height. In light of this, it can be claimed that the growing quails’ gut health was strengthened by the combination of EOs and probiotics. In a similar vein, Heydarian et al. [30] and Pipaliya et al. [31] documented enhancements in the histomorphology of villus in birds that have been given EOs and probiotics.

Our results demonstrate that nutritional supplements with EOs, probiotics, and a mixture of both positively affect organ development in quails. The combined use of EOs and probiotics exhibited the most significant improvements in carcass yield and spleen percentage, indicating a synergistic interaction. The spleen percentage showed notable differences, with the S_3_ group recording the highest value (0.23% ± 0.02). This suggests improved immune function due to the synergistic effects of EOs and probiotics, which are known for their antimicrobial and immunomodulatory properties. Probiotics enhance the gut-associated lymphoid tissue, which is closely linked to systemic immunity [26].

Nevertheless, blood tests could be applied to assess the health and wellbeing of birds alongside their physiological condition [32]. The quails fed a blend of EOs and probiotics had the most significant statistical value for TP and the highest numerical values for ALB and GLB, reflecting the results of the current protein profile. The combination of EOs and probiotics exhibited the greatest improvement in the serum protein profile due to their synergistic action. The same results were reported by Oleforuh-Okoleh et al. [33]. The two main proteins in blood, ALB and GLB, are crucial for feeding, metabolite delivery, and colloid equilibrium regulation. Likewise, GLB, also known as immunoglobulin, may represent an animal’s tolerance [34]. The effect of EOs and probiotic supplementation on protein metabolism may be related to improvement of intestinal amino acid absorption in acidic conditions that consequently enhances protein synthesis [35,36].

Gopi [37], who reported the effects of broiler chicks fed a ration containing EOs, found that the chicks’ TP was significantly higher. According to Paryad and Mahmoudi [38], chicks fed 1.5% yeast had higher plasma TP, ALB, and GLB concentrations, which elevate colloidal osmotic pressure in the blood and boost humoral mediated immunity. These results are in line with the current study’s findings. In a similar vein, among the experimental treatments, the growth promoters under study had a substantial impact on serum BUN content. In this study, the BUN level in the quail serum dramatically dropped in the supplemented groups, demonstrating that EOs and probiotics enhance quail protein synthesis, slow down the rate at which proteins decompose, and boost nitrogen use efficiency. This is most likely because EOs and probiotics can help animals grow and enhance feed conversion, which increases protein storage and feed digestibility and utilization [39].

As the quails were being fed EOs and probiotics, their lipid profiles were also assessed. In comparison to the quails on a regular diet, our findings showed that giving a diet enhanced with EOs or probiotics significantly reduced blood TG, TC, and LDL. However, other than the quails supplemented with a combination of EOs and probiotics, no change in HDL value was observed in the quails fed either EOs or probiotics. The current study supported the findings of Yalçın et al. [40], who noted that bolstering laying with yeast autolysate substantially lowered serum TC and TG levels. Gopi [37] additionally revealed that supplying a blend of oregano and cinnamon EOs notably diminished plasma TC and TG levels when compared to the untreated diet. Certain EO components block 3-hydroxy-3-methylglutaryl coenzyme A reductase, which is the enzyme that controls cholesterol synthesis [41]. The exact same results were also observed in research by Mahboobi et al. [42] and Wang et al. [43], who demonstrated that probiotic treatment considerably lowered serum TC levels. Moreover, Al-Saad et al. [44] analyzed the blood biochemistry of chickens fed probiotic mannan oligosaccharide; the results indicated a considerable drop in blood serum TC. These alterations in the aforementioned blood indices may help to explain the favorable impact of the intake of EOs and probiotics on the bodily functions of quail, which could then be related to the growth-promoting properties of these substances.

The elevated globulin concentrations linked to growth promoter treatments in this study might help explain the quails’ improved immunological tolerance. When compared to the quails fed a control diet alone, we discovered that supplementing the experimental quails with EOs or probiotics raised their serum levels of IgY and IgM. Interestingly, higher concentrations of Ig were seen in the treated quails given diets providing a combination of EOs and probiotics and probiotics alone. Probiotics have recently been shown to help raise the subject’s serum indices of IgY and IgM [45,46]. Yan et al. [47] and El-Shenway and Ali [48] proved that plant extracts improved immune response. However, other studies have found no effects of EOs on Ig levels [49,50]. An elevated serum Ig content serves to control and strengthen mucosal defense and immune regulation, which promotes quail health and performance, reduces oxidative stress, and offers other health benefits [51].

Oxidative stress can result from environmental stress, which can also speed up the generation of reactive oxidative species (ROS) [52]. In addition to causing a number of illnesses, oxidative stress can also have an impact on a bird’s growth rate, meat quality, and FCR [53,54]. In the meantime, prior research has proven that antioxidant enzymes can stop oxidative stress and lessen the formation of ROS [55]. Accordingly, incorporating natural antioxidants into poultry feed to boost the efficiency of antioxidant enzymes is essential for preventing lipid oxidation, bacterial translocation, and intestinal mucosal oxidative damage as well as for promoting the healthy growth and development of birds [56,57]. Our outcomes revealed that the incorporation of EOs and/or probiotics had an influence on improving the antioxidative condition of the studied quails, as the quails fed a combination of EOs and probiotics had the least amount of MDA, which is a significant byproduct of lipid peroxide, and the greatest T-AOC, SOD, and GPx in their blood compared to the other studied groups. In the same context, when the quails were supplemented with EOs and/or probiotics, there was a significant increase in antioxidant markers (SOD, CAT, GPX, and ATOX1). This improvement of antioxidant status in growing quails may partly explain the enhanced intestinal morphology. The unique function of EOs as a natural antioxidant has been validated by numerous investigations [58,59]. Utilizing natural plants and their derivatives was shown to increase oxidative balance in the bird’s body. Additionally, our findings supported those that showed probiotic administration can raise antioxidant enzyme levels, which were thought to be a key indicator of enhanced antioxidant defenses [60,61].

To additionally verify whether the growth-promoting effect of EOs or probiotics corresponds to an anti-inflammatory effect, the levels of TGF-β, IL-10, IL-1β, IL-6, and TNF-α were measured. The dietary EOs and probiotics anti-inflammatory beneficial properties in growing Japanese quail were confirmed by the noticeable rise in the levels of TGF-β and IL-10 and decrease in the levels of IL-1β, IL-6, and TNF-α. As acknowledged, the inflammatory suppressors TGF-β and IL-10 are crucial for establishing intestinal physiological equilibrium, limiting the generation of inflammatory arbitrators, and fostering both specific and natural immunity [62,63]. The three most significant pro-inflammatory cytokines—IL-1β, IL-6, and TNF-α—are essential for the initiation of inflammation, and high concentrations of them can cause harm to tissues [64], decrease growth hormone secretion [65], and alter protein and energy metabolism to impair growth performance [66]. According to Ocaña and Reglero [67], thyme oil components suppressed the overall release of pro-inflammatory cytokines, such as TNFα, IL1β, and IL-6. Furthermore, there is mounting evidence that the colonization of probiotics in the intestines decreases the production of pertinent pro-inflammatory cytokines and helps to create an anti-inflammatory condition [68]. Also, it has been researched that taking probiotic supplements raised IL-10 levels to assist [69]. Supplementing with dietary yeast hydrolysate reduced blood TNF-α levels and raised IL-10, which is in line with our findings [70]. In addition to the inflammatory indices listed above, probiotics and essential oils can be added to food to help birds’ anti-inflammatory health.

In this context, there was a notable increase in growth (IGF-I, myogenin, and AvUCP), immunological (IL-2, IL-4, IL-6, and AVBD), antioxidant (SOD, CAT, GPx, and ATOX1), and intestinal absorption (VEGF, MUC2, GLUT2, calbindin, and FABP6) markers in the quails supplemented with EOs and/or probiotics when compared to the control group. The combination of EOs and probiotics had the most noticeable impact on the markers’ expression patterns. To our knowledge, the combined effect of using EOs and probiotics on the expression profile of growth, immune, antioxidant, and intestinal absorption markers in quails is scarcely reported.

Kürekci et al. [19] found that quails fed EOs had considerably decreased expression of the avUCP gene but that the expression levels of the IGF-1 and myogenin genes in their muscle were unaffected. IGF-I generally stimulates amino acids, glucose uptake, enhanced DNA synthesis, tissue growth stimulation, and overall embryogenesis modulation to promote the proliferation of preadipocytes, chondrocytes, and fibroblasts [71]. Ojano-Dirain et al. [72] reported that avUCP contributes significantly to the regulation of poultry body weight gain by promoting energy dissipation through mitochondrial oxidation. One type of myogenic regulatory factor that controls muscle growth is the myogenic gene (MyoG) [66]. In his discussion of the relationship between MyoG polymorphism and the meat quality and slaughter characteristics of broiler chickens, Wang [73] discovered a strong positive link between MyoG and the development of chicken muscle fiber.

The preservation of intestinal integrity and function is thought to be crucial for birds’ optimal health and performance. Poultry intestine health is closely related to immune responses, gut function, and the digestion and absorption of nutrients [74]. In the current study, the quails fed with EOs and/or probiotics had their intestinal absorption assessed using VEGF, MUC2, GLUT2, calbindin, and FABP6 markers. It is important to note that FABP6 and MUC2 gene expression in the mucosa may serve as possible indicators for the health of the poultry gut barrier [75]. Through either independent mechanisms or aided diffusion via the GLUT2 in the basolateral membrane, glucose exits the cell and enters the circulation [76].

Probiotics can compete with pathogens for resources and adhesion sites, produce antibacterial compounds, and boost immunity, among other methods, to produce antimicrobial action [77]. Pelicano et al. [78] demonstrated that the intestinal mucosa of broilers given probiotics had a deeper crypt. According to a different study, probiotic supplements boost immunity and improve the length and surface area of ileal and jejunal villi, which encourages growth [79].

It has been demonstrated that supplementing with orange essential oil improves the digestibility of crude proteins [80] and chymotrypsin activity in the digestive system, which in turn stimulates the digestive system [81] to produce more digestive enzymes and improves the utilization of digestive products by improving liver functions [82]. Moreover, an increase in endogenous digestive enzyme secretions may be the cause of the improvement in FCR brought about by phytogenic feed additives, which also improves broiler gastrointestinal absorption and nutrient digestion [83]. Gut morphology was markedly enhanced by EOs [84]. In their research, Giannenas et al. [85] found that the height of the ileal and jejunal villi increased significantly when a combination of EO and benzoic acid was added to the diet. Immune cell morphologies and proliferation are impacted by the rise in immune tissue weight. The EOs’ thymol and carvacrol content is probably what caused this improvement [86]. It has been shown that the organosulfur components of EO trigger human neutrophil responses with ROS production, lymphocyte proliferation, and macrophage phagocytosis [87]. When comparing the quails supplemented with EOs and/or probiotics to the control group, the changes in the expression pattern of intestinal absorption markers may be explained by the previously indicated explanations.

## 5. Conclusions

This study provides the efficacy of EOs and probiotics on metrics like serum immunoglobulin levels, antioxidant activity, and overall health of quails. Their inclusion in quail diets improved live body weight, feed conversion ratio, immune-antioxidant status, and intestinal health, leading to enhanced nutrient utilization. Combining probiotics with EOs enhanced performance over individual treatments, indicating additive or synergistic effects that merit further exploration in future studies. The current findings should not be interpreted as definitive conclusions on the viability of EOs and probiotics as alternatives to antibiotic growth promoters (AGPs) but rather as indicative of their potential based on observed improvements when applied independently of AGP comparisons. Future studies incorporating AGP benchmarks are indeed recommended to robustly verify these findings and further substantiate the efficacy of alternative growth promoters in poultry raising.

## Figures and Tables

**Table 1:** Ingredients and nutrient composition of the basal diet.

Ingredients	Amount/kg		
Yellow corn	54.9		
Soybean meal	35.4		
Corn gluten	5.8		
Corn oil	0.8		
Limestone	1.5		
Dicalcium phosphate	0.7		
Premix *	0.3		
Salt	0.3		
DL. Methionine	0.1		
DL. Lysine	0.2		
**Items**	**Calculated amount**	**Analyzed amount**	
**Metabolizable energy (ME, kcal/kg)**	2906.68	2895.4	
**Crude protein (CP %)**	24.08	23.95	
**Ca%**	0.83	0.81	
**Available p, %**	0.54	0.52	

* The premix of vitamins and minerals utilized to meet the dietary requirements per kilogram of feed included the following concentrations: vitamin A (10,000 IU), vitamin D3 (1500 IU), vitamin E (10 mg), vitamin K3 (2 mg), vitamin B1 (2 mg), vitamin B2 (5 mg), vitamin B6 (3 mg), vitamin B12 (0.01 mg), niacin (27 mg), folic acid (1 mg), biotin (0.05 mg), pantothenic acid (10 mg), manganese (60 mg), zinc (50 mg), copper (10 mg), iodine (0.1 mg), selenium (0.1 mg), cobalt (0.1 mg), and iron (50 mg).

**Table 2:** The oligonucleotide primers used in real-time PCR, along with their sequence, accession number, annealing temperature, and PCR product size, were associated with genes involved in growth, antioxidant activity, and intestinal health.

Gene	Isolation Source	Primer	Product Length (bp)	Annealing Temperature (°C)	Accession Number
*IGF-I*	Liver	F: ACTCAAACTGTCCACACGCA R: AGCATCAGGGAGGTCCTTCT	155	58	XM_015867574.2
*Myogenin*	Muscle	F: ATGTCGCTCTCTGACCAACG R: CAATGGCACACAGCAGTCAG	76	60	XM_065747526.1
*AvUCP*	Muscle	F: TCCCCTGTCACTTCGTGGCCGC R: GTAGAGACCAGCGACACCGTCCT	170	60	BAK26782.1
*IL-2*	Spleen	F: GTGCAAAGTACTGATCTTCGCC R: CTTGGTGTGTAGAGCTCGAGATG	195	60	AY613440.1
*IL-4*	Spleen	F: GAGAGCATCCGGATAGTGAAG R: TTCGCATAAGAGCTGGGTTC	168	58	AB559571
*IL-6*	Spleen	F: CAACCTCAACCTGCCCAA R: GGAGAGCTTCCTCAGGCATT′	85	60	AB5595724
*AVBD*	Spleen	F: CTATTCGTGCTTGTCGACTTC R: CTTACACAGCAAGAGCTTCTT	106	58	AB698019.1
*SOD*	Liver	F: TGGACCTCGTTTAGCTTGTG R: ACACGGAAGAGCAAGTACAGR	126	58	NM_205064.1
*CAT*	Liver	F: CCTGACTATGGTGCGCGTAT R: CAGACACACGAGAAGTGGCT	106	58	XM_015863594.1
*GPx*	Liver	F: TTGTAAACATCAGGGGCAAA R: TGGGCCAAGATCTTTCTGTAA	140	58	NM_001163245.1
*ATOX1*	Liver	F: TTGTGGACATGACCTGCGAA R: AACTGGACACCTCCCAGTCT	73	60	XM_015875801.2
*VEGF*	Intestine	F: TCCGATTTTGAAAGCGGTGG R: CAATGCATCTCTTTCGCCGC	185	58	XM_015857736.1
*MUC2*	Intestine	F: CGACTCCATCAGAGTCACCG R: CGGAGTGGATGAGGATACGC	104	60	XM_032444897.1
*GLUT2*	Intestine	F: TGCATCTCAGACACACGTCC R: CCTGGCTCAGTGTGTTGCTA	140	60	XM_015872003.1
*Calbindin*	Intestine	F: CGAGATCTGGCACCACTACG R: TCGGGTGTTAAGTCCAAGCC	120	60	XM_015855985.2
*FABP6*	Intestine	F: TGGCAAGATCTCTTCTAGTCCAC R: GCACCACCTCGGTGACTATT	187	58	XM_032447429.1
*β-Actin*		F: CTGGCACCTAGCACAATGAA R: CTGCTTGCTGATCCACATCT	123	58	AF199488

**Table 3:** The effect of EOs, probiotics, or their combination on growth performance of growing quails.

Parameters	Groups				
	S_0_	S_1_	S_2_	S_3_	*p*-Value
IBW, g	49.5 ± 1.57	50.5 ± 1.38	51 ± 1.25	50 ± 1.67	=0.902
FBW, g	226 ^c^ ± 5.57	242 ^bc^ ± 5.07	249 ^b^ ± 5.42	270.5 ^a^ ± 5.8	<0.0001
BWG, g	176.5^c^ ± 4.09	191.5 ^bc^ ± 3.8	198 ^b^ ± 4.29	220 ^a^± 4.3	<0.0001
FCR	4.2 ^a^ ± 0.1	3.8 ^b^ ± 0.07	3.59 ^b^ ± 0.08	3.04 ^c^ ± 0.06	<0.0001

S_0_: control diet with no supplements, S_1_: control diet supplemented with EOs, S_2_: control diet supplemented with probiotics, S_3_: control diet supplemented with a combination of EOs and probiotics, a–b–c: values with distinct letters within the same row are significantly different (*p* < 0.05). Results are presented as means ± standard error (SE). IBW represents initial body weight, FBW represents final body weight, BWG represents body weight gain, and FCR represents feed conversion ratio.

**Table 4:** The effect of EOs, probiotics, or their combination on carcass characters of growing quails.

Parameters	Groups				
	S_0_	S_1_	S_2_	S_3_	*p*-Value
Live body weight	231 ^c^ ± 4.4	253 ^bc^ ± 7.26	263^ab^ ± 6.01	283 ^a^ ± 4.4	=0.001
Dressed carcass %	60.98 ^b^ ± 1.06	63.9 ^ab^ ± 0.55	64.07^ab^ ± 0.61	66.18 ^a^ ± 0.49	=0.006
Heart %	0.790 ± 0.02	0.813 ± 0.01	0.946 ± 0.10	0.784 ± 0.03	=0.222
Gizzard %	3.3 ± 0.27	2.8 ± 0.09	3.48 ± 0.19	3.18 ± 0.16	=0.117
Liver %	2.69 ± 0.04	2.4 ± 0.53	2.66 ± 0.19	2.69 ± 0.1	=0.872
Spleen %	0.15 ± 0.05	0.19 ± 0.03	0.14 ± 0.06	0.23 ± 0.02	=0.477

Different letters within the same row indicate significant differences (*p* < 0.05).

**Table 5:** The effect of EOs, probiotics, or their combination on blood protein and lipid profile of growing quails.

Parameters	Groups				
	S_0_	S_1_	S_2_	S_3_	*p*-Value
**Protein profile**					
TP (g/dL)	2.08 ^b^ ± 0.15	3.08 ^ab^ ± 0.42	3.14 ^ab^ ± 0.29	3.72 ^a^ ± 0.17	=0.0103
ALB (g/dL)	1.08 ± 0.11	1.33 ± 0.45	1.26 ± 0.40	1.38 ± 0.38	=0.942
GLB (g/dL)	1 ± 0.11	1.76 ± 0.07	1.88 ± 0.68	2.34 ± 0.51	=0.225
BUN (g/dL)	0.86 ^a^ ± 0.12	0.71 ^ab^± 0.12	0.65^ab^ ± 0.11	0.37 ^b^ ± 0.07	=0.049
**Lipid profile**					
TG (mg/dL)	125.58 ^a^ ± 4.27	107.42 ^ab^ ± 4.93	112.29 ^ab^ ± 2.46	93.89 ^b^ ± 5.73	=0.003
TC (mg/dL)	228.16 ^a^ ± 5.25	191.86 ^bc^ ± 4.89	210.49 ^ab^ ± 4	179.07 ^c^ ± 3.97	<0.0001
HDL (mg/dL)	58.76 ^b^ ± 4.28	65.90 ^ab^ ± 4.32	64.23 ^ab^ ± 5.12	78.49 ^a^ ± 4.42	=0.055
LDL (mg/dL)	114.26 ^a^ ± 4.32	96.96 ^ab^ ± 4.50	101.33 ^ab^ ± 4.29	83.20 ^b^ ± 4.44	=0.003

Different letters within the same row indicate significant differences (*p* < 0.05).

**Table 6:** The effect of EOs, probiotics, or their combination on blood immunity, redox state, and inflammatory status of growing quails.

Parameters	Groups				
	S_0_	S_1_	S_2_	S_3_	*p*-Value
**Immunity**					
IgY (mg/mL)	0.55 ^c^ ± 0.02	0.68 ^b^ ± 0.04	0.77 ^b^ ± 0.02	0.91 ^a^ ± 0.01	<0.0001
IgM (mg/mL)	0.25 ^b^± 0.008	0.28 ^ab^ ± 0.01	0.28 ^ab^ ± 0.01	0.33 ^a^ ± 0.02	=0.015
**Redox status**					
MDA (nmol/mL)	4.10 ^a^ ± 0.42	2.53 ^ab^ ± 0.41	2.73 ^ab^ ± 0.44	1.58 ^b^ ± 0.31	=0.006
T-AOC (U/mL)	7.60 ^b^ ± 0.92	9.88 ^ab^ ± 0.43	8.93 ^b^ ± 0.63	12.26 ^a^ ± 0.49	=0.002
SOD (U/mL)	40.84 ^c^ ± 1.87	57.30 ^b^ ± 1.96	51.09 ^b^ ± 2.68	70.55 ^a^ ± 2.29	<0.0001
GPx (U/mL)	64.73 ^b^ ± 3.79	74.18 ^ab^ ± 3.55	78.45 ^ab^ ± 3.93	86.20 ^a^ ± 3.33	=0.001
**Inflammatory response**					
IL-1β (Pg/mL)	54.50 ^a^ ± 2.90	43.25 ^ab^ ± 2.87	40.29 ^b^ ± 3.24	37 ^b^ ± 2.58	=0.006
IL-6 (Pg/mL)	78 ^a^ ± 2.86	64.25 ^ab^ ± 3.68	54.75 ^bc^ ± 3.97	46.25 ^c^ ± 2.53	=0.0001
TNF-α (Pg/mL)	269.75 ^a^ ± 3.94	261 ^ab^ ± 2.97	255 ^ab^ ± 3.92	248 ^b^ ± 3.63	=0.008
TGF-β (Pg/mL)	92.50 ^c^ ± 2.02	98.50 ^bc^ ± 2.25	107 ^b^ ± 2.35	117.75 ^a^ ± 1.49	<0.0001
IL-10 (Pg/mL)	28 ^c^ ± 1.83	35.25 ^bc^ ± 2.81	43.75 ^ab^ ± 2.84	51.50 ^a^ ± 3.88	=0.001

Different letters within the same row indicate significant differences (*p* < 0.05).

## Data Availability

Upon justifiable demand, the supportive data for the findings of the study will be provided by the relevant author.

## Abbreviations

The following abbreviations are used in this manuscript:

AGPs	Antibiotic growth promoters
ALB	Albumin
ATOX1	Antioxidant protein 1
AVBD	Avian beta defensin
AvUCP	Avian uncoupling protein
BUN	Blood urea nitrogen
BWG	Body weight gain
CAT	Catalase
CD	Crypt depth
cDNA	Complementary DNA
Eos	Essential oils
FABP6	Fatty acid binding protein 6
FBW	Final body weight
FCR	Feed conversion ratio
FI	Feed intake
GLB	Calculated globulin
GLUT2	Glucose transporter 2
GPx	Glutathione peroxidase
HDL	High-density lipoprotein
IBW	Initial body weight
IgY	Immunoglobulins
IgM	Immunoglobulins
IGF-I	Insulin-like Growth Factor 1
IL-1β	Interleukin-1β
IL-2	Interleukin-2
IL-4	Interleukin-4
IL-6	Interleukin-6
IL-10	Interleukin-10
LBW	Live body weight
LDL	Low-density lipoprotein
MDA	Malondialdehyde
MUC2	Mucin 2
NRC	National research council
SOD	Superoxide dismutase
T-AOC	Total antioxidant capacity
TC	Total cholesterol
TG	Triglycerides
TGF-β	Transforming growth factor beta
TNF-α	Tumor necrosis factor-α
TP	Total protein
VEGF	Vascular endothelial growth factor
VH	Villous height
VW	Villous width
